# Recruiting for the Training-Schools

**Published:** 1920-07-03

**Authors:** 


### THE MATRONS' AND SISTERS' DEPARTMENT.

### RECRUITING FOR THE TRAINING-SCHOOLS.

It is beginning to be evident that the plan of sitting still in an office and awaiting applications from candidates can no longer be relied on to fill the training-schools. The College of Nursing devoted one session of its recent conferences to this important matter, but, interesting as were the papers contributed on this occasion, it can hardly be said that much light was thrown on the problem of getting into touch with the women likely to make good nurses. It is all very well to declare that a high standard of preliminary education should be established and that girls should be encouraged to spend in colleges the year between leaving school and beginning their training, but the immediate need of many matrons is to find a sufficient number of women of average intelligence and normal health who will consent to sign on for three years. Certain celebrated training-schools can, no doubt, pick and choose and reject all candidates who have not received a liberal education. The vast majority must be satisfied with the existing level of education, which to our sorrow we must admit is far below that of some other countries. Until that is raised, not for one class only but for women in general, the hospitals must continue to train women whose education leaves much to be desired and must supplement deficiencies as best they may.

The shortage of probationers has had the effect of considerably improving the probationer's lot, not in pay and length of duty hours alone. So far, we have often pointed out, it has done nurses good service. But alas, while the disadvantages of the nursing career have been widely advertised, the improvements which have taken place are still unknown to that section of the public from which candidates are recruited. For the moment the pendulum has swung decidedly too much the other way, and nursing as a career has gone "out of fashion." It is probable, nay, certain, that it will swing back in time. But the need is urgent and we cannot afford to wait. Can nothing be done to bring the solid attractions of the nursing profession more forcibly before the women who are awaited vainly and impatiently by matrons working with depleted staffs?

Labour exchanges and such like central recruiting offices for particular occupations are just a little out of favour at the present moment. It might be a costly matter to institute a central bureau for receiving candidates for all the nursing services and advising them as to their career, but we believe this to be an essential feature in a completely organised nursing service. Maybe the College of Nursing has enough on its hands for the moment, but if in their new and spacious premises they could set aside a room for this purpose and invite young women to apply for information about the nursing career, it is tolerably certain they would be able to demonstrate the existence of many excellent candidates who under the present *laissez-faire* system never get within the hospital doors, and that the hospitals would be willing enough to pay all expenses incurred by thus recruiting probationers. If the experiment proved so successful as to demand in the end an independent organisation much would be gained.

In America, where the shortage of probationers is as bad as over here, some of the hospitals are engaging a recruiting nurse to give addresses wherever young women are gathered together. Something of this kind might be attempted in London and other towns, and one could name half a dozen societies and a dozen women's clubs where a talk about the fine opportunities afforded by nursing would be popular and likely to produce results. In Chicago a cinema film has been prepared showing various stages of hospital life and its social attractions. Would not this be a valuable aid in dispelling ignorant notions of the nurse's daily round fostered by sensational articles in the press? There is room too for letters in the press over locally well-known signatures explaining the conditions of the training-schools. The public needs education on this subject, and will not receive it unless trouble is taken to circulate correct information in an attractive form. It is not enough once to have brought the claims of the nursing service to the notice of schoolmistresses and heads of colleges. This should be done every year and up-to-date information kept steadily before all education authorities. Something in the nature of a publicity campaign ought to be set afoot to relieve the hospitals in the present scarcity. Once started it would, we are convinced, arouse immense interest throughout the country and bring in a great accession of strength.

In this, as in so many directions, the hospitals are all working in isolation each for their own hand. What is needed is co-operation, unity, impulsion from the centre. Where can this be found apart from the College of Nursing?

